# Tissue Elasticity as a Diagnostic Marker of Molecular Mutations in Morphologically Heterogeneous Colorectal Cancer

**DOI:** 10.3390/ijms25105337

**Published:** 2024-05-14

**Authors:** Anton A. Plekhanov, Dmitry S. Kozlov, Anastasia A. Shepeleva, Elena B. Kiseleva, Liubov E. Shimolina, Irina N. Druzhkova, Maria A. Plekhanova, Maria M. Karabut, Ekaterina V. Gubarkova, Alena I. Gavrina, Dmitry P. Krylov, Alexander A. Sovetsky, Sergey V. Gamayunov, Daria S. Kuznetsova, Vladimir Y. Zaitsev, Marina A. Sirotkina, Natalia D. Gladkova

**Affiliations:** 1 Institute of Experimental Oncology and Biomedical Technologies, Privolzhsky Research Medical University, 10/1 Minin and Pozharsky Sq., 603950 Nizhny Novgorod, Russia; 2 Nizhny Novgorod Regional Oncologic Hospital, 11/1 Delovaya St., 603126 Nizhny Novgorod, Russia; 3 Nizhny Novgorod City Polyclinic #1, 5 Marshala Zhukova Sq., 603107 Nizhny Novgorod, Russia; 4 Institute of Applied Physics of the Russian Academy of Sciences, 46 Ulyanova St., 603950 Nizhny Novgorod, Russia

**Keywords:** colorectal cancer, compression optical coherence elastography, tissue stiffness, morphological patterns, molecular analysis, driver mutation

## Abstract

The presence of molecular mutations in colorectal cancer (CRC) is a decisive factor in selecting the most effective first-line therapy. However, molecular analysis is routinely performed only in a limited number of patients with remote metastases. We propose to use tissue stiffness as a marker of the presence of molecular mutations in CRC samples. For this purpose, we applied compression optical coherence elastography (C-OCE) to calculate stiffness values in regions corresponding to specific CRC morphological patterns (*n* = 54). In parallel to estimating stiffness, molecular analysis from the same zones was performed to establish their relationships. As a result, a high correlation between the presence of *KRAS*/*NRAS*/*BRAF* driver mutations and high stiffness values was revealed regardless of CRC morphological pattern type. Further, we proposed threshold stiffness values for label-free targeted detection of molecular alterations in CRC tissues: for *KRAS*, *NRAS*, or *BRAF* driver mutation—above 803 kPa (sensitivity—91%; specificity—80%; diagnostic accuracy—85%), and only for *KRAS* driver mutation—above 850 kPa (sensitivity—90%; specificity—88%; diagnostic accuracy—89%). To conclude, C-OCE estimation of tissue stiffness can be used as a clinical diagnostic tool for preliminary screening of genetic burden in CRC tissues.

## 1. Introduction

Colorectal cancer (CRC) is the third most common cancer type in the world, with more than a million new cases reported annually and the second leading cause of cancer-related deaths [1]. High mortality is associated with tumor progression, which, in almost 50% of cases, leads to the dissemination of distant CRC metastases, which are most often localized in the liver, lungs, and peritoneum, often leading to the uselessness of surgery [2]. The latest European Society for Medical Oncology practice guidelines for the management of patients with metastatic CRC highlights current improvements and trends in molecular biomarker diagnostics [3]. Molecular diagnosis of driver mutations and microsatellite instability (MSI) of CRC is important due to the direct impact of genetic burden on the treatment efficacy [4,5]. For example, the identification of any of the above biological determinants is a negative prognostic factor for the use of monoclonal antibodies to the epidermal growth factor receptor in therapy [6]. The efficacy of using anti-HER-2 inhibitors, panitumumab-oxaliplatin, and cetuximab-oxaliplatin therapy in patients with wild-type (wt) RAS is higher than in mutant ones [7,8,9]. Despite the poor prognosis of CRC cases with an established *BRAF V600E* mutation, the potential of a new therapeutic strategy with a combination of encorafenib and cetuximab has been demonstrated, which significantly increases overall survival without compromising the quality of life [10]. The efficacy of nivolumab and pembrolizumab therapy for CRC cases and established MSI is higher than that of microsatellite stability (MSS) cases [11]; in addition, the identification of MSI is often associated with further genetic counseling and identification of Lynch syndrome [12]. All the above makes it important to provide high-quality molecular diagnostic analysis of CRC to ensure effective therapy and adequate patient management.

Most often, molecular analysis of CRC in clinical practice is indicated only for cases with distant metastasis for the purpose of direct adjustment of first-line therapy [13]. However, there are several biological and technical challenges to implementing more widespread molecular testing of CRC, such as intratumoral heterogeneity of the studied material [14], the complexity of the sample preparation procedure [15], high DNA fragmentation [16], insufficient quantity of cancer cells before DNA extraction [17], etc. In addition, despite the undeniable diagnostic accuracy of current methods of gene amplification and sequencing, it is worth emphasizing that they involve a significant investment of time and resources, which limits the widespread use of molecular testing in clinical practice [18]. Considering all these factors together, one way to increase the coverage of molecular diagnostics in a larger number of genetic burden CRC cases is to find a new additional method of preliminary screening [19]. Recent studies establish the connection between the characteristic morphology and the molecular portrait of CRC [20,21]. Some papers demonstrate the connection between the identification of poorly differentiated tumor clusters (solid patterns inherent in high-grade CRC) and *KRAS* mutations [22]/*BRAF* mutations [23]/MSS [24]; the involvement of *KRAS* mutations in the formation of solid CRC patterns has also been suggested [25], as well as the ability to predict MSI cases when evaluating histological images of CRC using machine learning technologies [26]. This strengthening of the connection between morphological and molecular cancer features increases the potential of using modern optical diagnostics methods (which differ from routine histological examination in the ease of implementation and the speed of obtaining information of tissue structure) for screening and identifying genetically altered CRC cases.

Compression optical coherence elastography (C-OCE) is a reliable technique for measuring the stiffness of biological tissues, which has recently been actively used in oncology [27,28,29]. C-OCE measurements demonstrate that tumor cells consistently have significantly higher stiffness, and, therefore, C-OCE can be used to identify the tumor node, its boundaries, and cancer molecular subtypes [30,31,32,33]. The phase-sensitive C-OCE is based on conventional optical coherence tomography (OCT) technology, where infrared light with a wavelength of 1310 nm is used to scan tissue [34,35]. The principle of C-OCE is to estimate the axial gradient interframe phase variation caused by applying uniaxial compressive pressure to the studied tissue [36]. C-OCE allows one to perform mapping of tissue stiffness with the resolution of about ~40–50 µm (at the level for detection of individual morphological structures and cell clusters) and has previously confirmed its efficiency for in vivo non-invasive label-free monitoring of experimental tumors in therapy [37,38,39], diagnostics morphology of human breast cancer [40], and endometrial cancer [41].

In our previous studies, we demonstrated the capabilities of C-OCE for highly sensitive imaging of the CRC tissue structure based on the differences in the elastic properties of individual morphological components [42,43]. This research is the next step toward C-OCE application to CRC screening in terms of understanding the relationship between molecular mutations in cancer and their elasticity/stiffness. We present a comprehensive study of individual morphological patterns of CRC cells using three methods—C-OCE, molecular analysis, and histology—where histological examination plays a connecting role between changes in stiffness values determined by C-OCE and the presence/absence of *KRAS*, *NRAS*, *BRAF* genes driver mutations and microsatellite status performed by molecular analysis. The aim of this research is to determine the relationship between cancer cell stiffness values and the presence of current clinically significant molecular mutations of CRC to justify the potential of using C-OCE as a tool for rapid preliminary diagnosis of CRC genetic burden.

## 2. Results and Discussion

**Figure 1:**
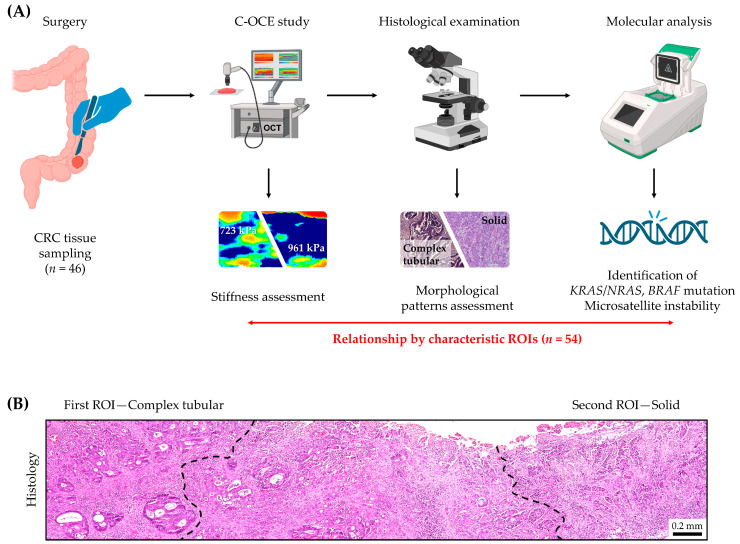
Schematic representation of study design. (**A**) The main steps: surgery and sampling of CRC tissue, ex vivo C-OCE study to evaluate elastic properties of fresh CRC samples, histological examination to identify the CRC morphological patterns, and subsequent molecular analysis of these patterns to detect the presence of gene alterations. (**B**) An example of delineation of two different morphological patterns (by two different regions of interest (ROI)) within one specimen. For two ROIs—complex tubular (left) and solid (right)—C-OCE and molecular analysis were separately performed, while the central tissue region with a mixed morphology was not assessed; scale bar size is shown in image.

CRC tissue samples (one from each patient) taken during CRC surgery were immediately transported to the C-OCE study site. After elasticity measurements, which took no more than 10 min for one sample, the tissues were sent for histological examination to identify characteristic morphological patterns in each case (Figure 1A). Subsequent targeted molecular analysis was performed for each morphological pattern, and results were compared with C-OCE data to identify the relationship between molecular mutations and tissue stiffness.

There is a generally accepted approach to the morphological classification of CRC tissue based on the identification of characteristic morphological patterns and their subsequent separate analysis [44,45]. These three base morphological patterns are as follows: (i) complex tubular pattern—glandular tumor structures, predominant in cases of low-grade colorectal adenocarcinoma; (ii) mucinous pattern—wide glands with fields of extracellular mucin, predominant in cases of mucinous adenocarcinoma; and (iii) solid pattern—non-glandular structureless accumulations of cancer cells, predominant in cases of high-grade colorectal adenocarcinoma. It is also known that several different morphological patterns (intratumoral heterogeneity) can occur within the same tumor, which requires separate molecular analysis [46,47]. Among the 46 samples of CRC tissue we analyzed, in 8 cases, two different morphological patterns were identified (Figure 1B). Therefore, the calculation of stiffness values and the identification of molecular mutations (driver mutations and microsatellite repeats) were carried out strictly in parallel in 56 regions of interest (ROI): 34 with complex tubular patterns, 6 with mucinous patterns, and 14 with solid ones. The transitional tissue regions consisting of mixed tubular/mucinous/solid patterns and tumor stroma were excluded from analysis (for details, see Materials and Methods, Section 3.3). A table with examined morphology for each CRC case is presented in Appendix A section, Table A1.

### 2.1. Stiffness Assessment of Colorectal Cancer Morphological Patterns

**Figure 2:**
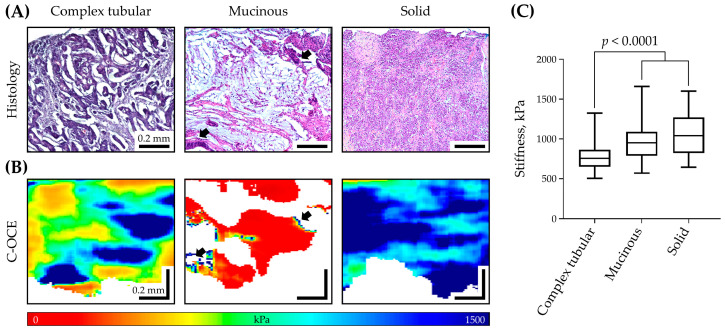
The results of C-OCE study of three CRC morphological patterns by ROIs: representative histological (**A**) and corresponding C-OCE (**B**) images for complex tubular, mucinous, and solid CRC morphological patterns; arrows in images of mucinous patterns indicate glandular cancerous structures—small areas of high stiffness values; scale bar size is shown in images. (**C**) Diagram of stiffness values distribution for three CRC morphological patterns. In the box plot, the central line represents the mean value, the upper and lower bounds indicate the standard deviation, and the whiskers indicate the minimum and maximum values; Mann–Whitney U test with Bonferroni correction was used to determine statistically significant differences.

A qualitative assessment of color-coded C-OCE images constructed for each of the three CRC morphological patterns (Figure 2A) demonstrated that each of them is characterized by certain predominant colors and distribution (Figure 2B). In particular, in C-OCE images, complex tubular patterns are characterized by inhomogeneous distribution of yellow, green, and blue zones corresponding to the glandular tumor structure; mucinous patterns are characterized by inhomogeneous distribution of small dark-blue zones among red and non-signal zones corresponding to the glandular tumor tissue among tumor stroma and extracellular mucinous fields; and solid patterns are characterized by more homogeneous (than for complex tubular) distribution of blue and dark-blue zones corresponding to the non-glandular cancer cell clusters.

Subsequent quantitative analysis of stiffness values was carried out only for C-OCE image zones with stiffness > 520 kPa (from yellow to dark-blue) because this threshold was previously established for identifying CRC cells [43]. Consequently, quantification of these three CRC morphological patterns by ROIs turned out to be quite different: complex tubular pattern had the lowest stiffness values (mean ± standard deviation) of 770 ± 164 kPa (Figure 2C), which were statistically significantly different from stiffness values of mucinous pattern of 968 ± 234 kPa and stiffness values of solid pattern of 1039 ± 270 kPa (*p* < 0.0001, Mann–Whitney U test). The stiffness values of mucinous and solid pattern areas were not statistically significantly different (*p* = 0.29). However, as was described above, visual C-OCE image assessment of these patterns allows for their distinction (Figure 2B), as we have shown previously in a paper [43].

This study continues our previous research on CRC using structural OCT methods [48,49] and ultrasound elastography [50]. Initially, diagnostic tasks in CRC were to differentiate CRC tissue from normal colon tissue and adenoma. Using structural OCT, it has been proven that attenuation coefficient mapping [51] and resorting to machine learning [52] allow for improving quantitative diagnostic results. Further, the application of ultrasound elastography has been successful in visualizing the depth of CRC invasion [53]. The combination of OCT and elastography in one technology—C-OCE—allowed getting closer to the CRC morphology and made it possible to distinguish the CRC morphological patterns of the tumor node [43]. In the current study, as well as in [43], CRC morphological patterns are also analyzed, but the groups include a larger number of ROIs, which allows for slight correction of the mean stiffness values for each of the CRC morphological patterns and proposes a stiffness value of 857 kPa for reliable differentiation of solid patterns from the complex tubular one.

### 2.2. Molecular Analysis of Colorectal Cancer Tissues and Its Correlation with Morphological Patterns

The frequency of occurrence of molecular mutations was analyzed for clinical cases (by grade of differentiation) as well as for ROIs in histological sections (by type of morphological pattern) (Table 1) [54].

For clinical cases, it was shown that somatic *KRAS*, *NRAS*, and *BRAF* driver mutations were identified in 44% of cases (20/46) (Table 1). Specifically, among them, in sixteen cases, activating mutations were identified in second (codons 12 and 13) and fourth exons (codon 146) of the *KRAS* gene. In one case, an activating mutation was detected in exon 3 (codon 61) of the *NRAS* gene, and in three cases, activating mutations were detected in exon 15 (codon 600) of the *BRAF* gene. A detailed table with the established molecular condition for each examined CRC case is presented in Appendix A section, Table A1.

The frequency of driver mutations in high-grade CRC cases was much higher (77%) compared to low-grade CRC cases in which driver mutations were significantly less common (30%) (Table 1). A statistically significant association (*p* = 0.0073, Fisher’s exact test) was established for the frequency of driver mutations occurrence with low differentiated high-grade CRC cases, which is consistent with the results of other studies [22,23,25]. It is also worth noting the prevalence of *KRAS* mutations (among all CRC cases) over *NRAS* and *BRAF*: *KRAS* mutations were found in every third case (35%).

Analysis of mutations in each CRC morphological pattern revealed the following dependence between the prevalent pattern and the kind of mutations: the solid pattern was more often associated with the occurrence of driver mutations (71%) than the complex tubular pattern (32%) (*p* = 0.0237, Fisher’s exact test). If the presence of complex tubular or solid patterns as markers of driver mutations is considered, then the diagnostic accuracy is 33% and 67%, respectively. By comparing the data of the high frequency of occurrence of driver mutations in solid CRC patterns and high stiffness values of these solid patterns established by C-OCE (Results Section 2.1), it can be concluded that C-OCE is expected to be effective in identifying CRC cases suspected of having a genetic burden. A detailed study of C-OCE efficacy in identifying genetically affected CRC cases and establishing a threshold stiffness value for the identification of such cases is described in the subsequent section (Results Section 2.3).

Regarding microsatellite repeats, a genetic alteration of MSI was detected in 23% of cases (3/13) of high-grade CRC, which may be associated with dysfunction of DNA mismatch repair [55] and indicates the progression of the tumor cells towards malignancy [24]. However, the identification of only three cases of MSI among the examined forty-six CRC cases does not allow us to speculate further on the relationship between MSI and stiffness of CRC cancer tissue, which will be addressed in future studies.

Molecular analysis by ROIs in histological sections revealed that somatic *KRAS*, *NRAS*, and *BRAF* driver mutations were identified in 44% of cases (24/54). In the solid pattern, these mutations were predominant (71% of cases) (Table 1). The frequency of occurrence of MSS was high (93% of cases), while MSI was identified only in 7% of cases. Case #21 needs to be considered separately, as heterogeneity in *KRAS G12V* mutation was detected. It was found to be present in the complex tubular pattern, but it was absent in the mucinous pattern. Intratumoral heterogeneity of CRC driver mutations similar to that established in the present study (case #21) was also noted in paper [56], where, in addition to a similar case of intratumoral morpho-molecular heterogeneity, the presence of *KRAS* mutations in different codons of different morphological patterns was detected using Next Generation Sequencing. In papers [57,58] on a par with intratumoral molecular heterogeneity, the intertumoral (between the primary tumor and metastases/recurrence) molecular heterogeneity of CRC was shown. There is a hypothesis that intra-/intertumoral heterogeneity is generated during the first few cell divisions on the way to cancer transformation, where the consequent clonal and subclonal alterations lead to the molecular heterogeneity of different tumor clones, which, due to uncontrolled rapid growth of the tumor, can often be located topologically in different areas of CRC tissue [59]. The ability to identify the tumor area most suspicious of the presence of mutations for subsequent molecular analysis is an urgent problem for prescribing effective therapy [60].

In the next section, we will examine the relationship between tissue stiffness and the occurrence of CRC molecular mutations to identify the effectiveness of C-OCE in the detection of tumor sites, which are the most suspicious for the presence of genetic burden.

### 2.3. Relationship between Tissue Stiffness and Molecular Mutation in Colorectal Cancer

**Figure 3:**
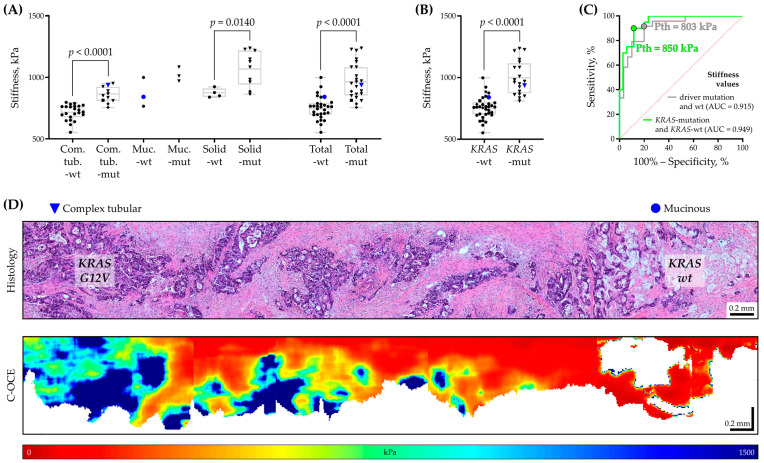
Comparison of tissue stiffness and the presence of CRC driver mutations. (**A**) Diagram of distribution of average stiffness values for CRC ROIs (for three CRC morphological patterns and total) depending on the presence or absence of driver mutations (*KRAS*/*NRAS*/*BRAF*). In the box plot, the central line represents the mean value, the upper and lower bounds indicate the standard deviation, the whiskers indicate the minimum and maximum values; each symbol is a mean stiffness value calculated by ROI in C-OCE image, the symbol shape indicates the following: triangle—*KRAS* gene mutation; square—*NRAS* gene mutation; diamond—*BRAF* gene mutation; dot—absence of any of the above driver mutations; symbols associated with case #21 are shown in blue. Mann–Whitney U test with Bonferroni correction was used to determine statistically significant differences. Designations: Com.tub.—complex tubular, Muc.—mucinous, wt—wild type, and mut—driver mutation. (**B**) Diagram of a distribution of average stiffness values for CRC ROIs with a *KRAS* driver mutation and *KRAS* wild-type. In the box plot, the central line represents the mean value, the upper and lower bounds indicate the standard deviation and the whiskers indicate the minimum and maximum values; each symbol is a mean stiffness value calculated by ROI in C-OCE image. (**C**) ROC curves showing the relationship between stiffness values and driver mutations: for a situation of presence/absence (wild type) of driver mutations (gray line), stiffness threshold (Pth) is equal to 803 kPa, AUC = 0.915; for a situation of presence/absence (wild type) of *KRAS* driver mutation (green line) Pth = 850 kPa, AUC = 0.949. ROC—receiver operating characteristic; AUC—area under ROC-curve. (**D**) Demonstration of the results of histological and C-OCE study for CRC case #21, where morphological intratumoral heterogeneity and corresponding heterogeneity in stiffness values were detected. The first ROI of complex tubular pattern (on the left side in images) with *KRAS G12V* driver mutation is characterized by high stiffness values, and the topologically separated second ROI of mucinous pattern (on the right side in images) without *KRAS* driver mutation (*KRAS* wild-type) has lower stiffness.

We aimed to further elucidate the relationship between stiffness values and the presence of specific molecular mutations. A comparison of stiffness values of each morphological pattern and of all patterns, along with the presence (mutant)/absence (wild type) of molecular mutations, is presented in Figure 3A. An overall statistically significant predominance of high stiffness values equal to 967 ± 145 kPa for CRC ROIs with driver mutations (*KRAS*, *NRAS*, and *BRAF* genes) was established over CRC ROIs without mutation (wild type), which have lower stiffness values equal to 751 ± 92 kPa regardless of morphological pattern type (*p* < 0.0001, Mann–Whitney U test). In addition, by analyzing stiffness values among each morphological pattern, a statistically significant predominance of high stiffness values also was established for CRC ROIs with driver mutations over CRC ROIs without mutations in groups of complex tubular (*p* < 0.0001) and solid (*p* = 0.0140) CRC patterns. Such regularity indicates a high degree of interconnection between the characteristic ‘presence of a driver mutation’ and ‘increased tissue stiffness’, which is confirmed by Mann–Whitney U test criteria. Here, it is worth recalling the previously established association between stiffness and molecular characteristics of breast cancer cells in successful C-OCE differentiation of morpho-molecular subtypes [32]. In this study, increased breast cancer stiffness was correlated with the absence of estrogen, progesterone, and epidermal growth factor HER2 receptors on or within cells. The results of other studies [61,62,63] assessing the mechanical properties of cells with molecular mutations using atomic force microscopy showed consistency with our results. In particular, activation of KRAS expression in human breast epithelial MCF-10A cells resulted in increased cell stiffness [61]. A similar increase in cell and nuclear stiffness was observed in cardiomyocytes carrying the *lamin A/C D192G* gene mutation compared to control (wild-type *lamin A/C* cardiomyocytes) [62]. It is worth highlighting the research [63] in which an increase in stiffness of the extracellular matrix in human CRC cells was detected with the *KRAS* mutation, while with the *BRAF* mutation, the stiffness did not change, which is quite natural with our results. Despite the inability of C-OCE to measure the stiffness of individual cells and stromal fibers due to the maximum spatial resolution of 40–50 µm, previously, we established a correlation between the different structural arrangements of collagen bundles (by multiphoton second-harmonic generation microscopy) and breast cancer tissue stiffness (by C-OCE) before and after chemotherapy [64]. Consequently, the results obtained in the current study and discussed above [61,62,63] confirm the existence of some relationship between changes in tissue stiffness and molecular mutations of CRC and encourage comprehensive research on this topic.

Next, we proposed two thresholds of stiffness values: first, above 803 kPa for separation of CRC ROIs with driver mutations of the *KRAS*, *NRAS*, and *BRAF* genes from the wild-type CRC ROIs; second, above 850 kPa for separation of CRC ROIs with a *KRAS* gene mutation form *KRAS* wild-type CRC ROIs (Figure 3C). These results indicate the effectiveness of C-OCE usage for rapid label-free targeted high-sensitivity determination of genetic burden in CRC tissues: in CRC ROIs with stiffness values above 803 kPa, the presence of one of *KRAS*/*NRAS*/*BRAF* driver mutations is detected with 91% sensitivity, 80% specificity and 85% diagnostic accuracy; CRC ROIs with stiffness values above 850 kPa the presence of a *KRAS* mutation is detected with 90% sensitivity, 88% specificity, and 89% diagnostic accuracy. According to our data, the diagnostic accuracy of identification of driver mutations by standard morphological analysis (morphological patterns) is 33–67% and by stiffness evaluation is 85%; hence, tissue stiffness appears to be a better predictor of molecular mutations than morphological analysis. Therefore, C-OCE could be an effective tool for preliminary screening of surgical samples of CRC tissue and predict the presence or absence of CRC driver mutations with ~85% diagnostic accuracy. In theory, such screening could significantly expand the coverage of molecular diagnostics to more cases of genetically altered CRC tissues and provide optimal treatment to more patients.

Regarding CRC case #21, where intratumoral molecular and morphological heterogeneity was discovered, stiffness values in CRC ROI of the complex tubular pattern were 937.7 kPa, which is higher than the established threshold stiffness value of 850 kPa for suspecting a *KRAS* mutation (Figure 3D). However, in the CRC ROI of the mucinous pattern, the stiffness values were 840.8 kPa, which did not exceed the threshold value. This CRC case demonstrates the possibility of C-OCE for intratumoral detection of the topology of the genetic burden cancer cell area. The fact that C-OCE predicts driver mutation in each of the examined CRC cases is more important to ensure subsequent targeted and complete molecular analysis. Thus, this study demonstrated the relationship between changes in cancer cell stiffness values and the presence of current clinically significant molecular mutations of CRC, which emphasizes the potential of testing C-OCE as a tool for the rapid preliminary diagnosis of CRC genetic burden in further larger studies.

However, some limitations of the presented study considering the prospects for using the C-OCE as a diagnostic tool in clinical practice need to be recognized: (i) the established diagnostic parameters require mandatory testing on a larger number of CRC cases; (ii) the implementation of C-OCE diagnostics is currently possible only in ex vivo study of CRC tissue for the following reasons: in compression C-OCE, direct access to the contact between the probe and the tissue being examined is needed, and there are no certified endoscopic options for C-OCE—they are still in the process of development and testing [43]; (iii) relatively small field of view (~4 × 1 mm) and required post-processing time per OCE image (~2 min) may significantly increase the time of examination of a large tumor node.

Nevertheless, the use of C-OCE can allow an express assessment of CRC tissue, enabling a reasonably high diagnostic accuracy in the detection of molecular mutations without the necessity of performing laborious and time-consuming conventional histology. Our C-OCE method can also be used as a guide for subsequent histological examination to verify the supposed most dangerous diagnostic conclusions. As a future step, one may foresee extensive and more accurate ex vivo tissue examination of a large number of CRC samples using C-OCE to have a big database. After that, machine learning can be implemented to answer the important question about mutation status as quickly and accurately as possible within a short time after tissue excision.

## 3. Materials and Methods

### 3.1. Colorectal Cancer Sample Characteristics

The study was conducted in accordance with the Declaration of Helsinki and approved by the Institutional Review Board of Privolzhsky Research Medical University (REB#3 of 21 February 2020). Informed consent was obtained from all participants. Human colon samples were collected from patients during surgery at the Volga District Medical Centre of Federal Medical Biological Agency of Russia.

The study included 46 patients with CRC: 33 low-grade and 13 high-grade. All the patients enrolled in the study had not been pretreated with radiotherapy or chemotherapy. From each patient, one sample of the center of tumor tissue was taken (Figure 1). Therefore, total number of studied samples was 46. The size of the samples varied from 15 mm to 27 mm. The samples were delivered to the laboratory in 10% BSA on ice within 30 min after resection and were studied within 30–70 min after the resection.

The histological examination included the identification of CRC morphological patterns—complex tubular, mucinous, and solid [44,45,65]. In 8 cases, two different morphological patterns were detected within one sample, so the total amount of considered regions of interest (ROI) was 54: complex tubular (*n* = 34), mucinous (*n* = 6), and solid (*n* = 14). All ROIs underwent parallel C-OCE and molecular analysis.

### 3.2. Compression Optical Coherence Elastography (C-OCE)

The C-OCE study was carried out using a spectral-domain multimodal OCT device (Institute of Applied Physics of the Russian Academy of Sciences, Nizhny Novgorod, Russia) with a central wavelength of 1310 nm, spectral width of 100 nm, and a receiving array enabling a 20 kHz rate of A-scan acquisition [66,67]. This device enables axial resolution of 10 µm, lateral resolution of 15 μm, and scanning depth of 2 mm in air. An advanced variant of phase-sensitive C-OCE [29,68,69,70] was used to visualize inter-frame strains in the tissue and subsequently map the Young modulus (tissue stiffness). Strain mapping was based on the estimation of axial gradients of interframe phase variations of the OCT signal using the ‘vector’ method [68,70].

It should be pointed out that the C-OCE study does not require any preliminary preparation of the samples. After being taken from the patient, the samples were quickly transported for C-OCE examination and the C-OCE data were immediately obtained. The time of one C-OCE scanning was ~30 s. Then, if necessary, the OCT probe was moved to implement the next measurement. On average, 4–6 C-OCE images were obtained from one sample. As a result, on average, after 10 min, the collection of C-OCE data was completed. Then, approximately 8–12 min were required to perform calculations and construct C-OCE images to see preliminary results of tissue stiffness in a specific case. Thus, C-OCE has a significant advantage in the imaging speed over histological examination.

During C-OCE scanning, an OCT probe was placed at the mucosa, so the study imitated an endoscopic examination of the colon lumen. Between the OCT probe and the studied surface of colon tissue, a reference silicone layer with pre-calibrated stiffness (with Young’s modulus 100 kPa) was placed. This allows for quantifying absolute values of Young’s modulus (kPa) for CRC tissue. The tangent elastic Young’s modulus of the tissue is defined as the ratio of incremental strain in silicone multiplied by silicone stiffness to the incremental tissue strain. In the resultant 2D C-OCE image, the resolution was about 4 times lower than in the initial OCT images, i.e., ~40–50 µm in both directions. For morphological analysis, this is not a drawback because this corresponds to the characteristic scale of ~10 cells, i.e., the scale on which the cells form a particular morphological component characterized by a specific stiffness value.

A key importance of the variant that was used for the C-OCE technique is that all C-OCE images are formed using a pre-selected pressure level (4 ± 1 kPa in the described study) standardized over the entire image area. The pressure standardization technique is based on the usage of the reference silicone layer as a sensor of local pressure, as described in detail in paper [71]. Without such standardization, the intrinsic elastic nonlinearity of cancerous tissues may result in uncontrollable variability of the estimated elastic modulus in different measurements and even different parts of the same image. The pressure-standardization procedures were critically important for enabling meaningful quantitative comparisons of elastographic data obtained from different measurements.

The so-obtained C-OCE images were represented in the color-coded form, such that stiffer areas are shown in blue, and soft areas are shown in red. Calculation of stiffness values was carried out only for C-OCE image zones with stiffness > 520 kPa (from yellow to dark-blue), as this threshold was established earlier for identifying CRC cells [43].

### 3.3. Histological Examination

Immediately after the C-OCE study, for co-location, the positions of the C-OCE scans were marked on the surface of the studied samples using histological ink (HistoLine, blue). Samples were fixed in 10% formalin for 48 h and then dehydrated using a gradient ethanol bath, followed by xylene purification and paraffin embedding. Then, several (3–6) serial sections with a 7 μm in thickness were made along the direction coinciding with the C-OCE-scan position. Histological sections were stained according to the standard technique with hematoxylin and eosin, which made it possible to assess the tissue microstructure (identification of grade and CRC morphological patterns) and to carry out an accurate comparison with C-OCE images [32,38,43].

### 3.4. Molecular Genetic Analysis

Molecular analysis was performed by microdissecting tumor tissue from unstained, recut slides of paraffin-embedded tumors. Areas for microdissection were selected by a pathomorphologist and circled on Hematoxylin and Eosin-stained slides for a microdissection template [56]. DNA was extracted by carefully scraping tissue from designated areas of slides with a clean blade and transferring the samples to separate tubes [72]. Genomic DNA was extracted using the QIAamp DNA FFPE Tissue Kit (Qiagen Inc., Chatsworth, CA, USA). The concentration of DNA was measured using the Qubit 4 (Invitrogen, Thermo Fisher Scientific, Waltham, MA, USA).

All samples were tested for somatic driver mutations of *KRAS*, *NRAS*, and *BRAF* genes, which are the most common in CRC [54]. *KRAS* exons 2, 3, 4 (12, 13, 61, 146 codons), *NRAS* exon 2, 3, (12, 13, 61 codons), and *BRAF* exon 15 codon 600 status were examined by two conventional methods of mutation detection.

First, the polymerase chain reaction (PCR) fragments were subjected to high-resolution melting analysis HRM, and abnormally melted fragments were examined by allele-specific PCR for the most common *KRAS*, *NRAS*, and *BRAF* mutations. PCR was carried out using EvaGreen chemistry in a final volume of 20 µL PCR reaction using CFX96 Touch Real-Time PCR System (Bio-Rad, Hercules, CA, USA). Reaction mixes were subjected to the following conditions: initial denaturation and polymerase activation at 95 °C for 10 min, followed by 50 cycles of denaturation at 95 °C for 15 s, annealing at 62 °C for 30 s, extension at 72 °C for 30 s. HRM was carried out and the data was collected over the melt range from 75 to 95 °C with 0.1 °C increment. After the PCR runs, the melting curve profiles were analyzed by Precision Melt Analysis™ v1.3 Software (Bio-Rad). To confirm the presence of mutations, PCR was performed using allele-specific primers.

For the analysis of MSI and amplification of the mononucleotide repeat marker BAT26, we used HPLC-purified Cy5 labeled forward and unlabeled reverse primers. PCR was carried out in a final volume of 20 µL PCR reaction using CFX96 Touch Real-Time PCR System (Bio-Rad). Reaction mixes were subjected to the following conditions: initial denaturation and polymerase activation at 95 °C for 10 min, followed by 5 cycles of denaturation at 95 °C for 15 s, touchdown annealing starting at 60 °C for 30 s (decreasing 1 °C per cycle), extension at 72 °C for 30 s, and an additional 33 cycles of denaturation at 95 °C for 15 s, annealing at 53 °C for 30 s, and extension at 72 °C for 30 s. The products were heated to 95 °C for 1 min and cooled to 40 °C for 1 min, allowing heteroduplex formation. After the touchdown PCR, 5 µL of each sample was mixed with 35 µL of Sample Loading Solution (Beckman Coulter, Brea, CA, USA) and 0.2 µL of DNA Size Standard-400 (Beckman Coulter). Samples were covered with mineral oil and analyzed on a CEQ 2000XL (Beckman Coulter) automated genetic analyzer. PCR products were visualized, and their size was estimated by using the CEQ 2000XL 11.0 Software Fragment Analysis Module (Beckman Coulter).

### 3.5. Statistical Analysis

The variables for statistical inter-group comparison were the stiffness values (Young’s modulus) calculated from C-OCE. Descriptive statistics results are expressed as mean  ±  standard deviation. Since this study includes a comparison of multiple groups with non-normal distribution data (Shapiro–Wilk test), the Mann–Whitney U-test with Bonferroni correction was selected. In all cases, the differences were considered statistically significant when *p* < 0.05 (the paper provides the exact *p*-values). Fisher’s exact test was selected for analysis of the association between the frequency of mutations and morphological features of CRC [73]. The assessment of the informative value and diagnostic capabilities of the studied method was carried out with an estimation of sensitivity, specificity, and diagnostic accuracy. Based on the sensitivity and specificity values, the receiver operating characteristic (ROC) curves were constructed, which show the dependence of the number of true positive rates on the number of false positive rates [74].

Statistical analysis was performed in the GraphPad Prism 8.0 (San Diego, CA, USA) and the Statistical Package for Social Sciences 26.0 (Chicago, IL, USA).

## 4. Conclusions

This study demonstrates the relationship between the stiffness values of CRC tissues and the identification of the current clinically significant molecular mutations. In cases of the presence of *KRAS*/*NRAF*/*BRAF* driver mutations, the stiffness values are higher than for wild-type (without gene mutations) cases. For the first time, the optimal threshold stiffness values for label-free targeted high-sensitivity identification of genetic burden CRC were established. Diagnostic testing established 91% sensitivity and 85% diagnostic accuracy of the C-OCE with a threshold of stiffness above 803 kPa for detecting the presence of one of *KRAS*, *NRAS*, or *BRAF* driver mutations in CRC, and 90% sensitivity and 89% diagnostic accuracy of the C-OCE with a threshold of stiffness above 850 kPa for detecting the presence of *KRAS* driver mutation in CRC. We believe that the use of C-OCE allows preliminary rapid, highly sensitive screening of CRC cases for the presence of genetic burden and the implementation of subsequent targeted molecular analysis.

## Figures and Tables

**Table 1:** Results of molecular analysis of CRC tissues with gradations by grade of differentiation (for clinical cases) and morphological patterns (for ROIs in histological sections).

		CRC Grade *			Morphological Patterns ^#^			
		Low- Grade	High- Grade	All Cases	Complex Tubular	Mucinous	Solid	All ROIs
**Driver** **mutations**	***KRAS***	21% (7/33)	69% (9/13)	35% (16/46)	24% (8/34)	50% (3/6)	64% (9/14)	37% (20/54)
	***NRAS***	3% (1/33)	-	2% (1/46)	3% (1/34)	-	-	2% (1/54)
	***BRAF***	6% (2/33)	8% (1/13)	7% (3/46)	6% (2/34)	-	7% (1/14)	5% (3/54)
	**Total**	30% (10/33)	77% (10/13)	44% (20/46)	33% (11/34)	50% (3/6)	71% (10/14)	44% (24/54)
	**Wild-** **type**	70% (23/33)	23% (3/13)	56% (26/46)	67% (23/34)	50% (3/6)	29% (4/14)	56% (30/54)
**Microsatellite repeats**	**MSI**	-	23% (3/13)	7% (3/46)	3% (1/34)	-	21% (3/14)	7% (4/54)
	**MSS**	100% (33/33)	77% (10/13)	93% (43/46)	97% (33/34)	100% (6/6)	79% (11/14)	93% (50/54)

*—proportion from all cases for this gradation group (number of cases/total number of cases). ^#^—proportion from all cases for this gradation group (number of ROIs/total number of ROIs).

## Data Availability

The data presented in this study are available upon request from the corresponding author.

## Appendix A

In the appendix section, an extensive table is presented, including the results of morphological analysis (CRC grade, Morphological heterogeneity, and Morphological pattern) and subsequent C-OCE stiffness assessment/molecular analysis of *KRAS*, *NRAS*, *BRAF* driver mutations and microsatellite repeats (MSR) status (Table A1).

**Table A1:** Results of morphological examination, C-OCE study, and molecular analysis for each examined CRC case.

Patient #	CRC Grade	Morphological Heterogeneity	Morphological Pattern	Average Stiffness (kPa)	Driver Mutation			MSR
					*KRAS*	*NRAS*	*BRAF*	
1	high	heterogeneous	Solid	1217.8	*A146T*	-	-	MSS
			Mucinous	1010.8	*A146T*	-	-	MSS
2	low	homogeneous	Complex tubular	866.4	*G13D*	-	-	MSS
3	low	homogeneous	Complex tubular	646.7	-	-	-	MSS
4	high	heterogeneous	Solid	1237	*G12D*	-	-	MSS
			Complex tubular	810	*G12D*	-	-	MSS
5	low	homogeneous	Complex tubular	718.3	-	-	-	MSS
6	low	homogeneous	Mucinous	764.5	-	-	-	MSS
7	low	homogeneous	Complex tubular	711	-	-	-	MSS
8	low	heterogeneous	Complex tubular	695	-	-	-	MSS
			Solid	838.7	-	-	-	MSS
9	low	homogeneous	Complex tubular	784.4	-	-	*V600E*	MSS
10	low	homogeneous	Complex tubular	796.3	-	-	-	MSS
11	low	homogeneous	Complex tubular	951	*G12D*	-	-	MSS
12	low	homogeneous	Complex tubular	835.3	*G12D*	-	-	MSS
13	low	homogeneous	Mucinous	998	-	-	-	MSS
14	low	homogeneous	Complex tubular	764.3	-	-	-	MSS
15	low	heterogeneous	Complex tubular	890.2	*G13C*	-	-	MSS
			Mucinous	1084.1	*G13C*	-	-	MSS
16	low	homogeneous	Complex tubular	850.6	*G12D*	-	-	MSS
17	low	homogeneous	Complex tubular	553	-	-	-	MSS
18	high	homogeneous	Solid	849.6	-	-	-	MSS
19	low	homogeneous	Complex tubular	772,7	-	-	-	MSS
20	high	heterogeneous	Solid	923	-	-	-	MSS
			Complex tubular	705.5	-	-	-	MSS
21	low	heterogeneous	Complex tubular	937.7	*G12V*	-	-	MSS
			Mucinous	840.8	-	-	-	MSS
22	high	homogeneous	Solid	863	*G13D*	-	-	MSS
23	low	homogeneous	Complex tubular	787.5	-	-	-	MSS
24	low	homogeneous	Complex tubular	758.3	-	-	-	MSS
25	high	heterogeneous	Solid	875.5	-	-	-	MSI
			Complex tubular	639.7	-	-	-	MSI
26	low	homogeneous	Complex tubular	755	-	-	*V600E*	MSS
27	low	homogeneous	Complex tubular	765.4	-	-	-	MSS
28	high	homogeneous	Solid	1148.4	*G12D*	-	-	MSI
29	low	homogeneous	Complex tubular	651	-	-	-	MSS
30	high	homogeneous	Solid	1082.8	*Q61L*	-	-	MSS
31	high	homogeneous	Solid	886	-	-	*V600E*	MSI
32	low	homogeneous	Complex tubular	677	-	-	-	MSS
33	low	homogeneous	Complex tubular	760.8	-	-	-	MSS
34	high	homogeneous	Solid	1227.6	*G12V*	-	-	MSS
35	low	homogeneous	Complex tubular	781.7	-	-	-	MSS
36	low	homogeneous	Complex tubular	812.8	-	*Q61X*	-	MSS
37	high	homogeneous	Solid	962.2	*G13D*	-	-	MSS
38	low	homogeneous	Complex tubular	614.7	-	-	-	MSS
39	low	heterogeneous	Complex tubular	924.4	*G13C*	-	-	MSS
			Mucinous	970.2	*G13C*	-	-	MSS
40	low	homogeneous	Complex tubular	702.7	-	-	-	MSS
41	high	homogeneous	Solid	982.8	*G12V*	-	-	MSS
42	low	homogeneous	Complex tubular	743.4	-	-	-	MSS
43	low	homogeneous	Complex tubular	734.7	-	-	-	MSS
44	low	homogeneous	Complex tubular	697.2	-	-	-	MSS
45	low	homogeneous	Complex tubular	761.6	-	-	-	MSS
46	high	homogeneous	Solid	1127	*G12D*	-	-	MSS
